# The cystic fibrosis pathogen Achromobacter xylosoxidans inhibits biofilm formation of Pseudomonas aeruginosa

**DOI:** 10.1099/jmm.0.002051

**Published:** 2025-08-01

**Authors:** Cecilia Sahl, Agnes Andersson, Natalie Larsson, Magnus Paulsson, Oonagh Shannon, Lisa I. Påhlman

**Affiliations:** 1 Division of Infection Medicine, Department of Clinical Sciences, Lund University, Lund, Sweden; 2 Wallenberg Centre for Molecular Medicine, Lund University, Lund, Sweden; 3 Clinical Microbiology laboratory, Skåne University Hospital, Lund, Sweden; 4 Section for Oral Biology and Pathology, Faculty of Odontology, Malmö University, Malmö, Sweden; 5 Division of Infectious Diseases, Skåne University Hospital Lund, Lund, Sweden

**Keywords:** *Achromobacter xylosoxidans*, biofilm, cystic fibrosis, polymicrobial interactions, *Pseudomonas aeruginosa*

## Abstract

**Background.** *Achromobacter xylosoxidans* and *Pseudomonas aeruginosa* are two pathogens that cause persistent airway infections in individuals with cystic fibrosis (CF). The persistence of *P. aeruginosa* is partly due to a high capacity to form biofilms and the ability to exert antagonism against other bacteria. Loss of microbial diversity in conjunction with chronic *P. aeruginosa* colonization is strongly correlated with low lung function in CF. *A. xylosoxidans* and *P. aeruginosa* are frequently co-isolated in CF airway cultures. This study aims to investigate the reciprocal effects on growth inhibition and biofilm formation between *P. aeruginosa* and *A. xylosoxidans in vitro*.

**Method.** Six isolates of *A. xylosoxidans*, isolated from three CF patients in early and late stages of a chronic infection, were cultured together with a CF isolate of *P. aeruginosa*. Biofilm formation was assessed using a microtiter assay and crystal violet staining. Quantitative PCR was used to quantify species proportions in biofilms. Growth curves were performed to compare planktonic growth rates.

**Results.** Three *A. xylosoxidans* isolates, all of which were from early-stage infections, inhibited biofilm formation of *P. aeruginosa*. The inhibition was concentration-dependent and required the interaction of live bacteria during the early stages of biofilm development. The inhibitory effect was not caused by nutrient depletion of the planktonic cells. The selected *A. xylosoxidans* isolate had a stronger capacity to adhere to plastic surfaces compared to the *P. aeruginosa* isolate.

**Conclusions*****.** A. xylosoxidans* can inhibit *P. aeruginosa* biofilm formation *in vitro*. The observed effect requires active interactions between live cells during the attachment stage of biofilm formation, possibly due to differences in adhesion capacity.

## Introduction

The microbiome composition of the cystic fibrosis (CF) airway correlates with the severity of lung disease, where a loss of microbial diversity and colonisation with certain bacterial species, including *Pseudomonas aeruginosa*, often indicates more severe outcomes [1,3]. The cystic fibrosis transmembrane regulator (CFTR) mutation in CF causes an accumulation of viscous mucus in the airways, which constitutes a permissive environment for bacterial and fungal growth. The infections lead to progressive airway damage due to primarily neutrophilic inflammation [4].

Biofilm formation is one of the main causes of persistent *P. aeruginosa* infections in people with CF [5]. By embedding within a matrix of polysaccharides, nucleic acids and proteins, bacteria can evade host immune responses and antibiotics. Once a chronic *P. aeruginosa* infection has been established, antibiotic treatment is used to reduce the bacterial load but is rarely sufficient to eradicate the pathogen [6], even after treatment with CFTR modulators that reduce mucus production. In addition to resisting host immune defences and antibiotic treatments, the capacity of *P. aeruginosa* biofilms to persist in the CF airway is also affected by its interactions with other species in the microbiome. It has been previously shown that *P. aeruginosa* employs multiple antagonistic mechanisms towards *Staphylococcus aureus*, the most common CF infection in early life, to gain a competitive advantage [7].

*Achromobacter xylosoxidans* is a Gram-negative bacterial species that is associated with decreased lung function and more frequent requirement for intravenous antibiotics and hospitalizations in people with CF (pwCF) [8,10]. Similarly to *P. aeruginosa*, these infections are difficult to eradicate once established and may persist for many years. In the respiratory tract, *A. xylosoxidans* undergoes adaptation and in-host evolution to increase its fitness in the niche [11,12]. Previous studies show that both *P. aeruginosa* and *A. xylosoxidans* are independently associated with increased inflammatory markers [13,14] and low lung function [15], but patients infected with both species have the worst outcomes [16]. In a large-scale screening study of interactions between the four CF pathogens *P. aeruginosa*, *Staphylococcus aureus*, *A. xylosoxidans* and *Stenotrophomonas maltophilia*, Menetrey *et al*. [17] show that only *A. xylosoxidans* was able to negatively impact the growth of *P. aeruginosa*.

In this study, we investigate interactions between clinical isolates of *P. aeruginosa* and longitudinal pairs of *A. xylosoxidans* isolated from the sputum of pwCF. The pathogens were grown in mono- and co-cultures to study both planktonic growth and biofilm formation with the aim of investigating competition between these pathogens throughout chronic infections and to determine the mechanisms involved.

## Methods

### Bacterial isolate selection

The isolates included in this study are presented in Table 1. Clinical isolates of *A. xylosoxidans* and *P. aeruginosa* from CF sputum cultures were obtained from the Clinical Microbiology laboratory, Skåne University Hospital, Lund, Sweden. Species identification was performed according to standard laboratory methods, including matrix-assisted laser desorption/ionization-time of flight MS. Species identification of *A. xylosoxidans* was further confirmed using whole-genome sequencing [11]. The *A. xylosoxidans* type strain KM543 was purchased from the Culture Collection University of Gothenburg. The *P. aeruginosa* reference strain PAO1 was a kind gift from Professor Arne Egesten, Lund University. All isolates were grown in Luria–Bertani (LB) broth (Sigma-Aldrich).

**Table 1.** Included bacterial strains

Isolate name	Species	Origin
PAO1	*P. aeruginosa*	Reference strain
PsA4	*P. aeruginosa*	CF sputum, Lund, Sweden
PsA9	*P. aeruginosa*	CF sputum, Lund, Sweden
KM543	*A. xylosoxidans*	Type strain, ear infection, Japan
AX-1A	*A. xylosoxidans*	CF sputum, Lund, Sweden
AX-1B	*A. xylosoxidans*	CF sputum, Lund, Sweden
AX-3A	*A. xylosoxidans*	CF sputum, Lund, Sweden
AX-3B	*A. xylosoxidans*	CF sputum, Lund, Sweden
AX-5A	*A. xylosoxidans*	CF sputum, Lund, Sweden
AX-5B	*A. xylosoxidans*	CF sputum, Lund, Sweden

### Biofilm formation and biomass quantification

Overnight cultures of each strain were normalized to OD_620_=0.6, diluted 1:10 in LB (18+162 µl) and grown in a 96-well plate. Co-cultures were prepared by mixing monocultures of *P. aeruginosa* and *A. xylosoxidans* in varying proportions (1:1, 2:1, 3:1 or 4:1), and 18 µl of the mixed suspensions were added to 162 µl LB in a 96-well plate. The plates were incubated for 72 h at 37 ℃ with 5% CO_2_ and 150 r.p.m. Wells were washed three times with PBS by pipetting to remove planktonic bacteria, and washed biofilms were then fixated with 200 µl of methanol for 10 min. The methanol was removed, and the plate was dried at room temperature for 3 h. 0.1% crystal violet was added in a volume of 200 µl, and the plate was incubated for 5 min. After washing the wells three times with PBS, the remaining stained biofilm was dissolved with 20:80 acetone–ethanol and diluted 1:3 before absorbance readout at 550 nm. Experiments were performed in biological and technical triplicate.

In separate experiments, PsA9 and AX-3A were grown in monocultures as described earlier. After 24 h of incubation, 18 µl of AX-3A was added to PsA9, 18 µl of PsA9 was added to AX-3A and bacteria were co-cultured for another 48 h. Alternatively, PsA9 was co-cultured 1:1 with AX-3A that had been heat-killed by incubation at 95 ℃ for 5 min or in the presence of 10% or 50% of cell-free AX-3A growth medium supernatant. The supernatants were prepared by growing AX-3A overnight in LB, pelleting the cells by centrifugation, followed by sterile filtering of the cell-free supernatants through a 0.22-µm syringe filter. After incubation for 72 h, biofilm mass was quantified using crystal violet staining as described earlier.

### Bacterial DNA quantification from planktonic bacteria and biofilms

Overnight cultures were centrifuged at 4,000 ***g*** for 5 min and resuspended to a concentration of ~2×10^9^ c.f.u. ml^−1^. Fifty microlitres of this suspension were added to 5 ml LB broth or 50+50 µl in co-culture. Cells were grown until reaching the stationary phase, ~24 h. 1.8 ml of the resulting culture was collected for homogenization using BeadBug (Benchmark Scientific), followed by DNA extraction using the DNeasy PowerLyzer Microbial Kit (QIAGEN) according to the manufacturer’s instructions.

Mono- or co-cultured biofilms, grown and washed with PBS as described earlier, were collected in 100 µl of PowerBead solution by scraping the wells with the pipette tip to dislodge the biofilms. Triplicate wells were pooled into one PowerBead tube before proceeding with homogenization and DNA extraction as described earlier. Extracted bacterial DNA was quantified with quantitative PCR (qPCR) using iTaq Universal SYBRGreen Supermix (Bio-Rad) using specific primers targeting *Achromobacter* spp.; AcForward: CACTAGCTCACGAACTCCAAGC, AcReverse: CAGCTTCAATCCTACCTAACTTTCCT [18] and *P. aeruginosa* rpoS; forward: TTGAGATACAGCTGCGTTGC, reverse: CTCCAAAAGCCACCACTTCC. Known concentrations of DNA from *A. xylosoxidans* or *P. aeruginosa* in tenfold dilutions were used as standards.

### Growth curves

Isolates were cultured overnight, diluted to an OD_620_ of 0.1 and added at a volume of 15 to 150 µl medium in a 96-well plate. The plate was incubated at 37 °C with 150 r.p.m. shaking and OD_620_ measurements were performed once every hour for 24 h using a SpectraMax M2 plate reader (Molecular Devices). In addition, growth rates were assessed in the presence of 10% or 50% bacterial cell-free supernatants. Growth rate experiments with supernatants were performed in 10-ml tubes containing 125 µl overnight culture (OD_620_ 0.1), 500 µl (10%) or 2,500 µl (50%) sterile-filtered supernatant, and fresh LB at a total volume of 5 ml.

### Adhesion

Overnight cultures of each isolate were normalized to an OD_620_ of 0.1. In total, 1 ml of bacterial culture (1 ml of monoculture or 0.5 ml of each species in coculture) was added to 12 well-plates. After incubation at 37 °C with shaking for 1.5 h, the growth medium containing non-adherent bacteria was removed, and the wells were washed once with PBS. DNA was extracted by the addition of 300 µl PowerBead solution (Qiagen DNeasy PowerLyser Microbial Kit, QIAGEN) to each well, and bacteria were detached with a cell scraper. Samples were transferred to PowerBead Tubes for homogenization and subsequent DNA extraction according to the protocol provided by the manufacturer. *A. xylosoxidans* and *P. aeruginosa* DNA were quantified using qPCR as described earlier.

## Results

### Evaluation of biofilm formation in *P. aeruginosa–A. xylosoxidans* cocultures

**Fig. 1.**
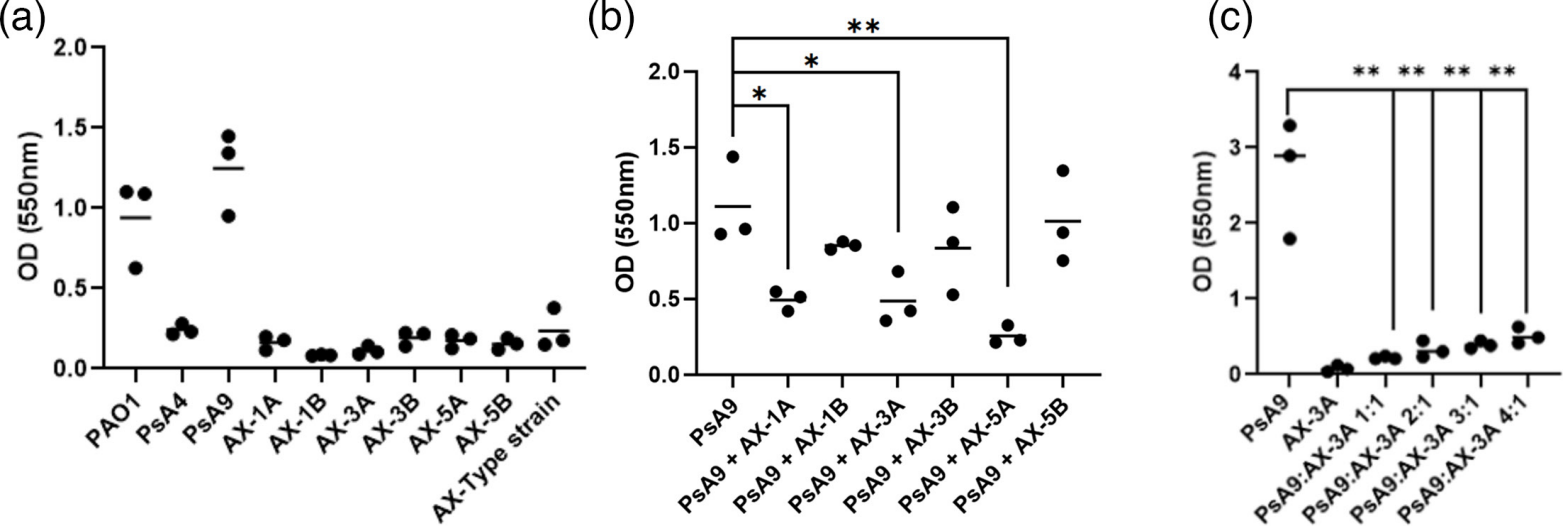
Biofilm formation of CF pathogens in mono- and co-culture. (**a**) Crystal violet quantification of biofilm formation of *P. aeruginosa* and *A. xylosoxidans* isolates in monoculture. (**b**) Biofilm formation of *P. aeruginosa* isolate PsA9 grown in monoculture or together with three pairs of early (AX-1A, AX-3A and AX-5A) and late (AX-1B, AX-3B and AX-5B) *A. xylosoxidans* isolates. (**c**) PsA9 was co-cultured with increasing proportions of *A. xylosoxidans* AX-3A, followed by quantification of biofilm formation. Each dot in the graphs represents the average value of 3 replicates, and bars represent the mean. *P<0.05, **P<0.01.

Biofilm formation is an important virulence mechanism for the establishment of chronic infections in the CF airway. We first screened two clinical *P. aeruginosa* and six clinical *A. xylosoxidans* CF isolates for biofilm formation using a crystal violet assay. In addition, type strains of *P. aeruginosa* and *A. xylosoxidans* were included as a reference. The clinical *A. xylosoxidans* isolates were longitudinal pairs obtained from the same patient at an early and a late stage of chronic infection (Table 2). Isolates were selected based on the *P. aeruginosa* colonization status of the corresponding patients. The first pair (1A-1B) was derived from a patient who was not infected with *P. aeruginosa*. The second pair (3A-3B) originated from a patient who originally had a persistent *P. aeruginosa* infection that was subsequently cleared during *A. xylosoxidans* infection. The third pair (5A-5B) was isolated from a patient with persistent *P. aeruginosa* infection throughout both the early and late stages of *A. xylosoxidans* infection. In the initial screening of biofilm formation, the *P. aeruginosa* isolates PAO1 and PsA9 were strong biofilm producers, whereas PsA4 and all *A. xylosoxidans* isolates tested were poor biofilm producers (Fig. 1a). To further investigate biofilm formation in dual-species biofilms of *P. aeruginosa* and *A. xylosoxidans*, we selected the strong biofilm-producing isolate PsA9 to study the effects of the different *A. xylosoxidans* isolates on biofilm formation. When PsA9 was co-cultured with the early isolates AX-1A, AX-3A and AX-5A, a significant reduction of *Pseudomonas* biofilm mass was observed compared to PsA9 grown in monoculture. In contrast, no biofilm reduction was seen when PsA9 was co-cultured with the late isolates AX-1B, AX-3B and AX-5B (Fig. 1b). To further characterize biofilm production in co-cultures, we focused on the interaction between AX-3A and PsA9 since this isolate had out-persisted *P. aeruginosa* during clinical infection (Table 2). First, we added AX-3A in varying proportions to investigate whether the observed effect was concentration-dependent (Fig. 1c). The biofilm-inhibiting effect decreased with lower concentrations of AX-3A. However, a significant inhibition of biofilm formation was still observed at a *P. aeruginosa* vs. AX-3A ratio of 4:1 (Fig. 1c).

**Table 2.** *Achromobacter xylosoxidans* CF isolates

Patient	Early *A. xylosoxidans* isolate	Colonization at the time of early isolation	Interval (years) between early and late isolates	Late *A. xylosoxidans* isolate	Colonization at the time of late isolation
CF 1	AX-1A	*A. xylosoxidans, Staphylococcus aureus*	6.8	AX-1B	*A. xylosoxidans*
CF 3	AX-3A	*A. xylosoxidans, P. aeruginosa, Staphylococcus aureus, Stenotrophomonas maltophilia*	3.6	AX-3B	*A. xylosoxidans*
CF 5	AX-5A	*A. xylosoxidans, P. aeruginosa, Staphylococcus aureus, Stenotrophomonas maltophilia*	7.2	AX-5B	*A. xylosoxidans, P. aeruginosa, Staphylococcus aureus, Stenotrophomonas maltophilia*

### Biofilm inhibition by *A. xylosoxidans* is caused by interactions between live bacteria during the early stages of biofilm formation

**Fig. 2.**
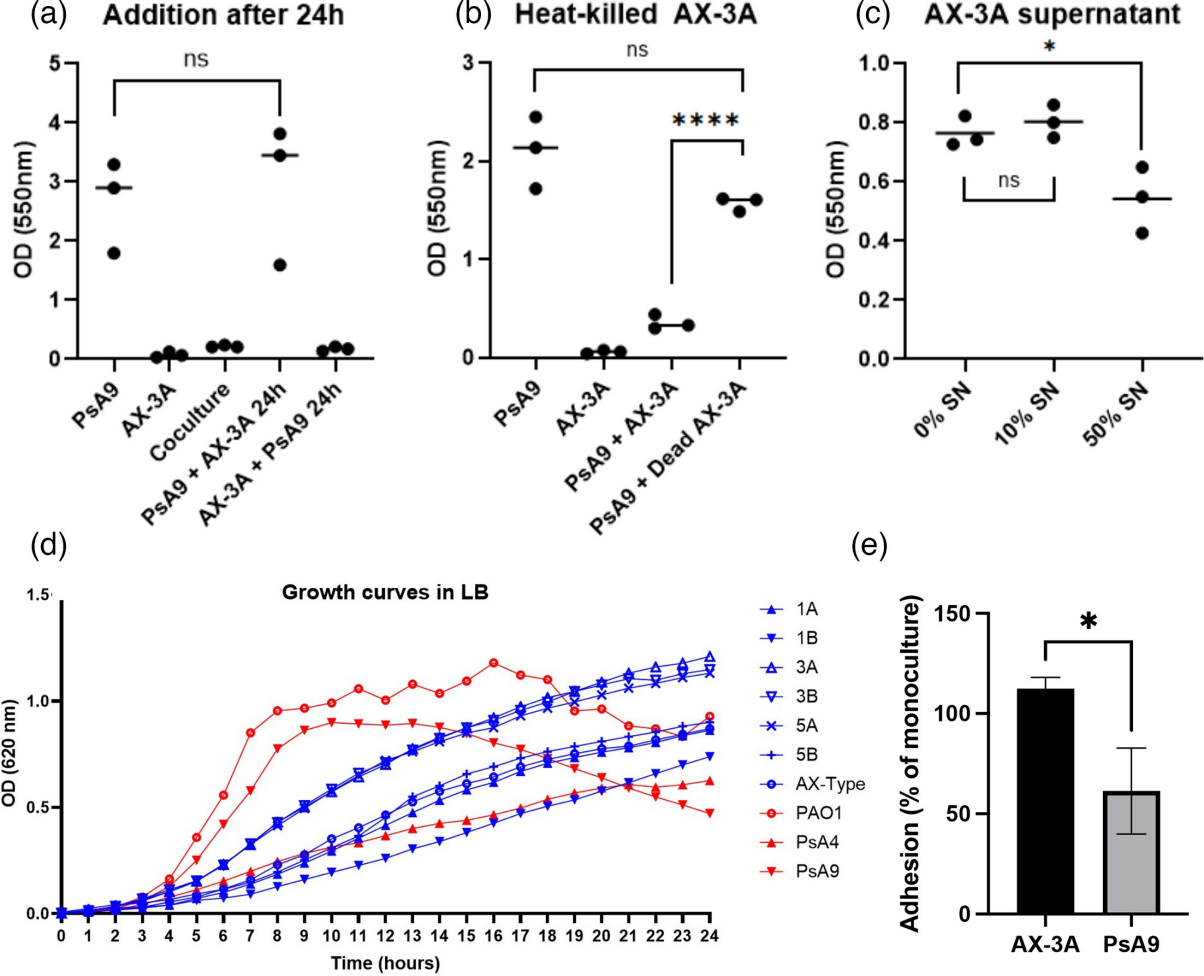
Mechanisms of interaction between *P. aeruginosa* and *A. xylosoxidans* during biofilm formation. (**a–c**) PsA9 and AX-3A were grown in dual- or single-species biofilms in 96-well microtiter plates for 48 h. (**a**) Addition of AX-3A to an established PsA9 biofilm after 24 h does not affect biofilm formation, and PsA9 cannot establish a biofilm in wells already colonized by AX-3A. (**b**) Co-culturing of live PsA9 with heat-killed AX-3A. (**c**) PsA9 was allowed to form biofilm in the presence of 0%, 10% or 50% of cell-free culture supernatant from AX-3A. (**d**) Growth curves of seven *A. xylosoxidans* and three *P. aeruginosa* isolates grown as planktonic monocultures in 96-well microtiter plates during 24 h with continuous hourly OD measurement. (**e**) Comparison of bacterial adhesion to microtiter plates. Equal volumes of PsA9 and AX-3A were incubated in microtiter plates for 90 min to allow bacterial adhesion to the wells. Monocultures were incubated in parallel for comparison. After washing steps, attached bacteria were detached, and *A. xylosoxidans* and *P. aeruginosa* DNA were quantified using qPCR. The graph shows bacterial DNA from adhered bacteria in co-cultures compared to monocultures in three replicates. All statistics are unpaired t-tests, *P≤0.05, ****P≤0.0001, ns, not significant.

Quantification of PsA9 and AX-3A DNA in co-culture biofilms revealed that the biomass of the dual-species biofilms was dominated by *P. aeruginosa*, but in lowered quantity compared to in parallel monocultures (Fig. S1A, available in the online Supplementary Material). A similar domination by *P. aeruginosa* was seen in dual biofilms with PsA9 and AX-3B (Fig. S1A). To study the mechanisms of competition by *A. xylosoxidans*, we next established a biofilm by culturing PsA9 or AX-3A for 24 h, after which planktonic AX-3A or PsA9 was added for the remaining 48 h of incubation. Adding *A. xylosoxidans* to an existing *P. aeruginosa* biofilm had no inhibitory effect on the biofilm mass. In contrast, PsA9 could not establish a biofilm in wells already colonized by AX-3A (Fig. 2a), indicating that the inhibitory interaction takes place during the establishment phase and not as a disruption of the *P. aeruginosa* biofilm. Next, we determined that heat-killed AX-3A did not affect biofilm production of PsA9 (Fig. 2b), indicating that the interaction requires live bacteria. Adding secreted *Achromobacter* products by incubating PsA9 together with 50% sterile-filtered supernatant of AX-3A moderately lowered the biofilm production (Fig. 2c), but not as strongly as live bacteria.

### Biofilm inhibition is not caused by differential growth rates

To investigate whether biofilm inhibition was caused by *A. xylosoxidans* outcompeting *P. aeruginosa* in growth kinetics, we analysed the planktonic growth rates of the bacterial strains included in the study (Fig. 2d). PsA9 was the most rapidly growing strain with the steepest slope during the exponential phase. None of the *A. xylosoxidans* strains displayed rapid growth during the exponential phase, indicating that biofilm inhibition was not caused by *A. xylosoxidans* outgrowing *P. aeruginosa*. To confirm that the observed inhibitory effect was not predominantly caused by nutrient depletion, we performed growth curves of PsA9 grown in 10% and 50% AX-3A supernatant. PsA9 did not exhibit growth inhibition in the presence of either concentration of AX-3A supernatant (Fig. S1B). Finally, we performed DNA quantification of broth co-cultures to investigate whether inhibitory effects also occur in planktonic cultures. After growth in liquid culture media until the stationary phase, the final DNA concentration of planktonic PsA9 was not lowered by the presence of AX-3A (Fig. S1C), indicating that the reduced biofilm formation observed in biofilm co-cultures was not caused by growth inhibition of PsA9.

### AX-3A exhibits stronger adhesion ability than PsA9

As planktonic *A. xylosoxidans* cultures did not grow faster than PsA9, we speculated that the interaction causing biofilm inhibition takes place at the early adhesion stage. To investigate adhesion ability, PsA9 and AX-3A/AX-3B in mono- or co-cultures were allowed to adhere to microtiter wells for 90 min. After washing, attached cells were detached, and bacterial DNA was quantified to assess the bacterial load. All three isolates adhered to a similar extent when incubated in monocultures (Fig. S2A). By comparing DNA concentrations from co-cultures versus monocultures, we found that a significantly lower proportion of PsA9 DNA was retrieved from co-cultures compared to AX-3A, suggesting a lower adhesion rate for PsA9 (Fig. 2e). In contrast, no significant difference was observed between PsA9 and AX-3B when grown in co-culture (Fig. S2B). Taken together, the data suggest that AX-3A negatively impacted PsA9 biofilm formation due to a higher adherence rate to the plastic of the microtitre well.

## Discussion

In this study, we report that *A. xylosoxidans* is capable of inhibiting biofilm formation by *P. aeruginosa*. We demonstrate that the interaction takes place during the early stages of biofilm development and is likely related to how efficiently the strain adheres to a surface. These findings are consistent with previous reports showing that approximately half of *A. xylosoxidans* strains possess this inhibitory capacity against *P. aeruginosa* biofilms [19]. Notably, all three isolates causing biofilm inhibition in our study were obtained from early-stage infections, whereas the corresponding late-stage isolates from the same patient lacked this ability. This observation aligns with earlier work demonstrating that *A. xylosoxidans* undergoes adaptive changes during chronic infections in CF airways, resulting in attenuation of virulence [11,12]. Similar persistence mechanisms have also been reported for *P. aeruginosa* [20]. In a previous study that included all three AX strains examined in the present work, *A. xylosoxidans* became less motile, slower growing and more resistant to antibiotics over time during chronic infection of CF airways [11]. Whole-genome sequencing revealed that mutations frequently occurred in transcriptional regulators, including loci associated with biofilm formation. Whether these genetic changes are relevant for inter-species interactions remains to be determined.

The clinical impact of these microbial interactions warrants further investigation. One of the key pathogenic features of chronic *P. aeruginosa* infection is its ability to dominate the lower respiratory microbiota, thereby reducing microbial diversity [1,21]. Similarly, *A. xylosoxidans* is also capable of dominating the bacterial community and can reach over 90% relative abundance [22]. Given that both pathogens appear to be equally capable of causing inflammation and lung injury in CF [13,15,23], a replacement of *P. aeruginosa* by *A. xylosoxidans* would not necessarily lead to improved clinical outcomes. Importantly, co-infection with both pathogens has been associated with lower pulmonary function in people with CF compared to mono-infections with either *A. xylosoxidans* or *P. aeruginosa* [16]. This observation raises the question of whether co-localization of the two species affects bacterial virulence. In experimental models of oral biofilms, both biofilm formation and the expression of virulence-associated genes were altered in *Streptococcus mutans* when co-cultured with other oral pathogens [24]. Investigating virulence factors other than biofilm formation in *A. xylosoxidans–P. aeruginosa* co-cultures may provide further insights into the clinical impact of these interactions.

A key limitation of this study is the potential discrepancy between *in vitro* observations and *in vivo* biofilm formation within the CF airways. Specifically, bacterial adhesion mechanisms in the lung environment may differ markedly from those observed in the microtiter well. Notably, previous studies have demonstrated competition on agar between *P. aeruginosa* and *A. xylosoxidans* in terms of growth, motility and pigmentation [17], suggesting that factors other than adhesion to microtiter wells are involved. Additionally, bacteria may respond differently to the *in vitro* growth conditions used in this study compared to the lung environment, where they may be under pressure from host defences, antibiotics and other microorganisms. Future studies could incorporate more *in vivo*-like conditions, such as using artificial CF sputum medium or investigating the effects of antibiotic pressure on microbial interactions. Furthermore, it would be of interest to select a biofilm-producing strain of *A. xylosoxidans* to study whether the inhibition may also be exerted by *P. aeruginosa*. Another technical limitation is the phenotypic similarity of *A. xylosoxidans* and *P. aeruginosa* on agar plates, as well as their shared resistance to commonly used antibiotics. These features prevented the use of c.f.u. counts and selective agar to quantify growth in co-culture experiments. Instead, we used qPCR and species-specific primers to estimate bacterial load via DNA quantification.

Given the increasing threat posed by antibiotic-resistant micro-organisms, bacterial biofilms are an important target for the development of novel therapeutic strategies [25,27]. The observed inhibition of *P. aeruginosa* biofilms by *A. xylosoxidans* warrants further investigation to identify specific molecular mechanisms involved. Such insights could improve our understanding of interspecific interactions and potentially identify new therapeutic targets against biofilm-associated infections, especially in the context of CF airway infections.

## Supplementary material

10.1099/jmm.0.002051 Uncited Supplementary Material 1.

## Abbreviations

CF: cystic fibrosis
CFTR: cystic fibrosis transmembrane regulator
LB: Luria–Bertani
pwCF: people with cystic fibrosis
qPCR: quantitative PCR
