# Supplementary material for: The cystic fibrosis pathogen Achromobacter xylosoxidans inhibits biofilm formation of Pseudomonas aeruginosa
Source: J Med Microbiol. 2025 Aug 1;74(8):002051. doi: 10.1099/jmm.0.002051 (PMC12316436; doi:10.1099/jmm.0.002051)
Supplement: Uncited Supplementary Material 1. [file jmm-74-02051-s001.pdf]

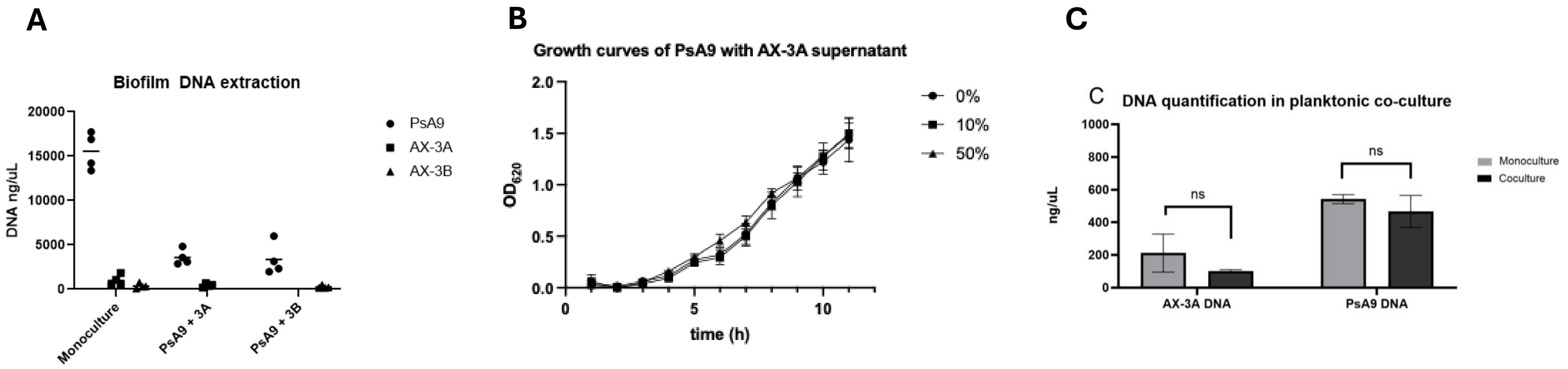

Supplementary figure 1. (A) Quantification of *P. aeruginosa* and *A. xylosoxidans* DNA from PsA9, AX-3A and AX-3B in monoculture and co-culture biofilms. (B) Growth curves of PsA9 in the presence of 0%, 10% or 50% AX-3A cell-free culture supernatant. (C) Quantification of *P. aeruginosa* and *A. xylosoxidans* DNA in planktonic mono- and co-cultures of PsA9 and AX-3A in LB broth at stationary phase.

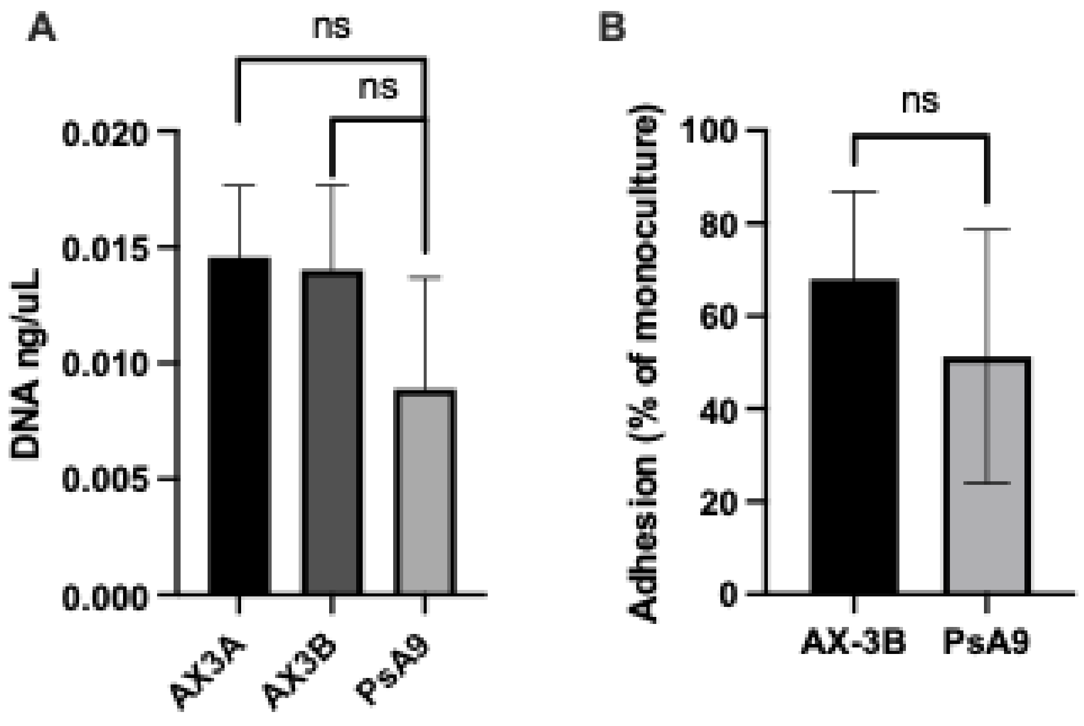

Supplementary figure 2: Comparison of bacterial adhesion to microtiter plates. Equal volumes of PsA9 and AX-3A or AX-3B were incubated in microtiter plates for 90 min to allow bacterial adhesion to the well. Monocultures of the two species were incubated in parallel for comparison. After washing steps, attached bacteria were detached and bacterial DNA was quantified using qPCR and specific primers for *A. xylosoxidans* and *P. aeruginosa*. (A) DNA-concentrations from adhered cells grown in monocultures. (B) Data from co-cultures were compared to results from the corresponding monocultures. The graph show bacterial DNA from adhered bacteria in co-cultures compared to monocultures in three replicates. ns=non significant.
